# A general theory of consciousness III the human catastrophe

**DOI:** 10.1080/19420889.2024.2353197

**Published:** 2024-05-26

**Authors:** Abraham Peper

**Affiliations:** Department of Biomedical Engineering & Physics, Academic Medical Centre, University of Amsterdam, Amsterdam, The Netherlands

**Keywords:** Afterlife, cognition, consciousness, cruelty, free will, history, inner speech, language, morality, punishment, religion, sensory images, the law, verbal communication, violence, visual thinking

## Abstract

It is generally assumed that verbal communication can articulate concepts like ‘fact’ and ‘truth’ accurately. However, language is fundamentally inaccurate and ambiguous and it is not possible to express exact propositions accurately in an ambiguous medium. Whether truth exists or not, language cannot express it in any exact way. A major problem for verbal communication is that words are fundamentally differently interpreted by the sender and the receiver. In addition, intrapersonal verbal communication - the voice in our head - is a useless extension to the thought process and results in misunderstanding our own thoughts. The evolvement of language has had a profound impact on human life. Most consequential has been that it allowed people to question the old human rules of behavior - the pre-language way of living. As language could not accurately express the old rules, they lost their authority and disappeared. A long period without any rules of how to live together must have followed, probably accompanied by complete chaos. Later, new rules were devised in language, but the new rules were also questioned and had to be enforced by punishment. Language changed the peaceful human way of living under the old rules into violent and aggressive forms of living under punitive control. Religion then tried to incorporate the old rules into the harsh verbal world. The rules were expressed in language through parables: imaginary beings - the gods - who possessed the power of the old rules, but who could be related to through their human appearance and behavior.

## 1. Introduction

The present state of mankind, the world with all the rules and laws man has created during his desperate pursuit of a peaceful coexistence, has an origin. This origin does not lie in ancient primitive civilizations, as is often assumed. The fundamental roots of society lie in the way man managed the transition from life as a normal animal to life as a creature who is ruled by his language.^1^ Much that is written about prehistoric man overlooks the important fact that he still showed many characteristics of animal behavior. The development of verbal communication changed his behavior and his development abruptly, as I will argue, and the history of men over the last 200,000 years is strongly affected by the confrontation between the old animal instincts and the effects of verbal communication.

As said, before language evolved, man was in many ways still an animal, and he behaved the way he had behaved for millions of years. But his behavior changed fundamentally when he began to communicate through language. In order to understand the behavior of presentday man, it is necessary to understand what happened then, how language could change a fairly normal animal into the often cruel and violent creature who now rules the planet: Homo lingua, the language man.

## 2. Recapitulation of the theory

### 2.1. Language and thought

When verbal communication developed, the way humans lived changed fundamentally. To understand how and why that change occurred, it is important to realize what the consequences of verbal communication were and still are for human behavior, and especially how the use of human language deforms the thought process. In order to make these effects clear, I will recapitulate the essence of my previous papers on language and consciousness [1,2], supplemented with new considerations and understanding of verbal communication and the effects of language especially relevant to the present discussion.

Fundamental to any understanding of what happened when language started to influence human life is insight into the phenomenon of human language itself: its deficiencies and its troublesome relationship with the thought processes in the brain. As was discussed extensively in my earlier research [2], verbal communication as a method of exchanging information is fundamentally flawed; any communication by language is inaccurate and questionable. The sense of consciousness about our own ideas expressed in spoken language suggests that we know what we were thinking. However, a verbally communicated thought does not reflect the preceding cognitive process in a very accurate way.

### 2.2. Consciousness and thinking

There has always been a search for understanding consciousness [3–11]. This paper propounds that consciousness in a living organism, cannot be understood. It is what biological sensory information evokes, and that’s all there is to it. For all practical purposes, consciousness is what the senses incite when they make their efforts known to the living organism they are part of. This paper holds that consciousness is a function of the senses; it is the result of what senses do, down to cell level [1,2,12–16].

My theory is based on what consciousness does and what the effects of thinking are, where thinking is the way an organism solves a problem through a neural process [1]. The phenomenon of sensory consciousness makes the outcome of that process known: the neural information is translated into conscious sensory images [1,2,17–22].

Thinking is a neural process and the outcome of such a process is neural data. For an animal, understanding this neural information is achieved by relating it to the outer world that initiated the cognitive activity. The senses supply the cognitive system with knowledge about the state of the environment. The cognitive system then processes that information to solve problems posed by the environmental situation. To make the outcome of neural cognitive processes understandable, it is translated into sensory images, known from relevant situations in the past (see [1]: the outcome of a cognitive process is translated into a composite of sensory images, together expressing the cognitive message (see e.g. [23,24]. These sensory images are, as said, fundamentally conscious and basically show the organism how the solution to the problem found by the cognitive system must be implemented if it is to be executed.^2^

### 2.3. Verbal communication

The natural way animals (and pre-language infants) become conscious of what they think, is by a transformation of the outcome of the neural cognitive process into conscious sensory images, as discussed in previous papers. Many of these images are inborn, but most originate from situations the animal encounters during its life while the congenital images are liable to change in response to environmental situations. The sensory images capturing the animal’s thoughts are thus completely personal, which has important consequences.

All animals communicate thoughts and intentions to others, but the extent of this communication is limited. Humans developed a method to communicate the outcome of almost all their personal thought processes to others. They do this by translating the sensory images which make the neural thought process conscious into series of sound pieces, which are then decoded by the listener and expressed in personal sensory images.^3^ Theoretically, this method has unlimited potential: all personal thoughts can – again, theoretically – be expressed in unique sound combinations for communication. In actuality, this method has many flaws and restrictions, as pointed out in my previous paper. Fatal for the method of verbal communication is that the sensory images of the sender and those of the receiver are personal, and therefore fundamentally different [2]. The words used in verbal communication will thus always be differently interpreted by the sender and the receiver.

Language is a system of signs which communicates a thought by referring to sensory images in the listener, assumed to correspond to the sensory images the thought evoked in the speaker. However, the neural cognitive process and the sensory mechanism are fast and complex, while language is an extremely slow and inaccurate method of coding information, as discussed in-depth previously [2]. Whereas the visual images of the natural process are 2- or even 3-dimensional, language is 1-dimensional: what is a complex neural cognitive activity is encoded into language tokens which are positioned sequentially as a string of symbols and somehow must express something comparable. The outcome of this operation deviates significantly from the neural cognitive information,^4^ while the conversion process itself is a tedious one.^5^ The fundamental problem is that thoughts cannot be expressed accurately in language. In addition, after the problematic conversion of neural cognitive information into language in the speaker, the verbal message has to be converted back into conscious sensory images in the listener. As the latter process is also an indeterminate transformation, comparable to that in the speaker, it distorts the message even further [2].

Language, or rather the use of words, is in itself not conscious; consciousness pertains to the sensory mechanism. The words are signs referring to sensory images, and it is these images which are conscious [2]. The sound of words is of course heard consciously, but the sound is only an intermediary, a tool to excite sensory images associated with the message. Hence, verbal consciousness, i.e. consciousness evoked by the words used in talking, is essentially sensory consciousness, similar to the natural conscious experience.

To summarize, the outcome of the neural cognitive process is transformed into sensory images, the meaning of which is translated into language for communication, after which it is translated back into sensory images in the listener to become conscious. It will be clear that these intermediate verbal interventions seriously limit and deform the message. Verbal communication yields a much impoverished version of the original neural thought.

### 2.4. Verbal thought

Additional to interpersonal verbal communication, verbal humans express their thoughts – the outcome of the cognitive process – in verbal communication to themselves. Instead of relying on the natural process of translating cognitive activity into sensory images, most humans translate their thoughts into audible language: the voice in their head (see e.g. [25]. This has most of the disadvantages of verbal communication and the result is that what becomes conscious in humans of their thoughts are deformed and often inconsistent versions of their thoughts.

The above rather peculiar phenomenon probably develops in children during the period the child learns language, after which it remains the way they think. My previous paper states [2]: *That humans talk to themselves is one of the most remarkable aspects of the use of language. It is so common that nobody experiences it as being strange; it is apparently seen as what humans do. But talking is communication and when humans turn their thoughts into spoken language, this is necessarily part of a communication process. And as communication is fundamentally between a sender and a receiver, there has to be somebody who listens when a person talks to himself. […] When a child starts talking to itself during the learning process, it communicates with an imaginary person, somebody it makes up, but who really feels alive (see also [26–28] much like the child imagines the teddy bear it plays with and talks to being alive. And when the child has grown up, that imaginary person is still there, somebody the adult talks to when he thinks and to which he also attributes his feelings and his actions.*

This intrapersonal verbal communication – inner speech – is a superfluous extension to the thought process and leads to significant distortions. The thought process in visual thinkers, who fundamentally do not use language while thinking, is incomparable more detailed and efficient than inner speech. Compared to the natural sensory mechanism, language is a simple medium. Sensory images are necessarily of a complexity comparable to that of the neural cognitive process as they developed in conjunction during the evolution of the human brain. If the sensory information is converted into language, this seriously deforms the thought process as language cannot handle the information content of the cognitive message very well and the translation will never be exact or even adequate [2]. Verbal thinking results in misunderstanding our own thoughts; verbally communicating a thought in any exact way, also to ourselves, is elusive. Furthermore, the cognitive process in verbal thinkers will generally anticipate the problems presented by the use of language and adapts to the limited possibilities language offers [2,18,29], resulting in an even worse outcome.

An additional consequence of thinking verbally is that the natural sensory way of becoming conscious of thoughts is scarcely accessible anymore as it has largely become unconscious. What is left of our natural way of thinking are our feelings, which cannot be accurately translated into language in any way [1].

Thinking in language distorts the train of thought fundamentally: any potentially adequate thought is always deformed by the restricted capacity of language to handle complex information. A verbal thinker will never know what his neural thoughts themselves have been: the translation into language determines what becomes conscious of his thoughts. This is the cause of the often incalculable and unpredictable outcome of the cognitive process. Analyzing our own thoughts when there exists an important deviation between what is thought and what becomes conscious must necessarily yield a poor result.

Unfortunately, most people are more or less verbal thinkers, with all the drawbacks discussed above, though there are numerous well-known visual thinkers,^6^ like Einstein, Wittgenstein and Sartre, to name a few. But they all use language too, although at different levels of excellence. It is not that there is a distinct separation between visual and verbal thinkers. There is a spectrum along which the senses combine with verbal thinking in varying degrees.

## 3. History of verbal communication

### 3.1. The impact of language on human life

When verbal communication developed to the extent that discussion and argumentation became possible, its unreliable and ambiguous nature must have had a major impact on human life. Verbal interaction must have been extremely confusing and troubling and must have led to much misunderstanding. In the following, I will try to analyze what might have happened then and what the effects were when human life changed from a natural animal state into in a verbal world.

The origin of language is largely unknown. This is necessarily so as any knowledge about how language developed is speculative, derived from animal or anatomical data [30–34]. Still, some observations can be made which are not speculative. Two important non speculative observations are that (1) at a certain moment in the evolution of language, its use changed from mere communication of information to analysis and discussion, and that (2) at one time, the overt verbal communication was supplemented with covert talk - the voice in our head. When these events happened we do not know, nor how long the transformations took to develop, but that they happened is certain and it seems clear that they influenced human behavior. Before the above changes in language use occurred, language was a tool which only supplemented the existing communicative abilities of humans; it did not change their functioning fundamentally. When verbal analysis and discussion developed, the life of humans changed dramatically.

### 3.2. Rules of behaviour

Before humans started using verbal communication, their behavior did not differ fundamentally from other animals. They had their culture like all animal species have their culture: their way of behavior, which is more or less identical for all individuals of a species [35–42]. The behavior of an animal in an animal culture - generally a species - is determined by what I will call ‘its rules’. By rules, I mean all that determines the behavior of a species in its natural habitat. These rules are followed by all animals of a species, without much deviation. They make that species what they are: a duck has the rules that make it a duck with its typical duck behavior; a cat has the rules that determine its special feline characteristics. The rules of a species are the solutions it found for the problems it faced during its evolution. Although important parts of the rules of a species are acquired after birth, it is important to realize that the rules of a species are fundamentally innate. This has important consequences for human behavior, as we will see. It is not so that all rules of different species are necessarily different. They often overlap or have characteristics in common. Pre-language man had his rules like all other animals, until he developed communication via spoken language and his behavior changed unrecognizably.

## 4. The transition to spoken language

When humans developed verbal communication, the rules as had existed almost indefinitely were called into question. With spoken language, any subject could be discussed, but discussions inevitably cause differences of opinion (see [2]. When, in those early times, humans discussed the use or function of their rules, the rules must have appeared blurred and, as they could not be articulated very well in spoken language, their value and usefulness could not be determined. But the most important effect of using language was that it was possible to discuss the rules at all and that their nature and use could be questioned. And as discussion seldom results in definite agreement, the effect must have been that there developed differences of opinion about their interpretation. The rules then lost their authority; they did not survive scrutiny through spoken language. The rules worked as long as everybody accepted them as unconditionally binding. As soon as doubt about their value and usefulness developed, the rules disintegrated and lost their power.

When the rules lost their absolute value and power, they were replaced by rules developed with spoken language. However, those new rules did not have authority and would not be followed. The fact that the old rules were challenged implies that the new rules were also disputed. I found three possible ways in which the transition from life under the old rules to a society based on rules developed through language could have taken place. 1. Man developed language based rules alongside the old rules, which then gradually disappeared while there was a peaceful cohabitation of the old and new rules. 2. The old rules were gradually abandoned and one by one replaced by language rules. 3. The old rules were analyzed in language and then abandoned after which there followed a period without any rules. In this case, the transition toward a society based on language rules happened considerably later. The gradual and peaceful options of 1 and 2 are not very likely. A change to life under new rules cannot happen without causing problems. As is the case in all animals, living under the old rules must have been natural, comforting and satisfying. The only reason to change the rules must have been the impact of language on the thinking of humans. But the development and then substitution of functioning language rules for the old rules, which encompassed all life, must have taken considerable time. Hence, the effect of using language must have been a period without effectively working rules. And without rules of how to live together, total chaos must have evolved. A species without rules cannot exist. The species is its rules. Rules are what makes it function. A species without rules is lost as a species and, at that time, homo as a species must have been lost. What emerged from the chaos was the talking human: a violent, cruel animal without natural rules, who desperately tried to live together following artificial rules, rules developed using ambiguous, ill functioning spoken language. When we look at the society we live in now and see how difficult it is to create and maintain rules, we can get an impression of the struggle early humans must have had to develop rules that would keep the species alive.

## 5. Religion

The chaos which followed the disappearance of the rules must have been enormous and must have lasted very long. It could even be maintained that chaos still exists. While the rules governing animals are completely binding - there are no animals who do not comply with the rules of their species - the laws humans contrived were not binding in practice and had to be enforced. However, the effect of enforcing those new rules was limited and they were often ignored, as I will argue below.

It is not the case that the old rules were lost completely. They lost their absolute power, but in an indirect way the rules kept working, as they still do to a certain extent. They show for instance in the empathic feeling humans can have towards other humans, which is an innate quality (see e.g. [43]), although it can disappear due to circumstances (see e. g. [44]). ^7^ Many remnants of the original rules can be found if we look for them. But they are no longer binding as they have largely been substituted by rules developed with language.

When the old rules were lost, humans suffered a total lack of understanding of their life and environment; without the clarity of the old rules, everything became incomprehensible. Explanations in language never sufficed and were always questioned. These feelings of incomprehension and the emptiness they created were then partly met by a new idea, a concept that still had some resemblance to the feeling and function of the old rules: religion, the gods. Instead of believing in the rules that had always governed man, people sought safety and clarity of life through a translation of the old rules into language metaphors. That which was still felt from the old rules was expressed in language through parables; the old rules were translated into imaginary beings who possessed the power of the old rules, but who could be related to through their human appearance and behavior [45].

The old rules were transformed through spoken language into a form which to a certain level allowed them to keep functioning: the gods. The gods were supposed to possess powers which could force humans to follow the old rules. Whether the emergence of religion was due to an expression of the remains of the old rules, or whether it was a more or less rational act by some who recognized the problems which developed when the rules disappeared cannot be determined, but both factors may have played a role. Either way, religion based its appeal on feelings the old rules could still evoke. And as the old rules represent basic human feelings, they must have been the basis of later religions too.

## 6. The gods and the old rules

It is generally assumed that the gods and religion arose from the lack of understanding prehistoric man had for the world he lived in. Man could not yet think very well and turned the things around him into symbols of something powerful that ruled the world [45–48]. However, as argued above, religion was a reaction to the chaos that resulted when the old rules were no longer followed. Religion tried to give the old rules a function again in the new reality created by language. It was not that man began to think when he developed language, as is often assumed. What happened was probably more or less the opposite: he lost all understanding of his world with it. With language, his life had suddenly become incomprehensible; with language a thing was given a name and was then lost as an intelligible concept. The gods – beings who resembled humans but had absolute power, just as the old rules were totally commanding – then reintroduced a kind of clarity in life, somewhat resembling the old, prelanguage way of living.

In language, complex subjects are expressed in a way that suggests those subjects are clear and understandable. However, the world is too complex to be expressed in such a defective and unreliable medium as language and, when language began to determine life, this created severe difficulties. Religion provided some comfort, but an important problem that emerged was death.

The old rules could not help make death understandable in language. Natural death for an animal is something which happens and which it accepts because death is a natural part of its life. For animals, the future does not exist in the way it does with talking humans. A hamster saves food for the future, but not because it worries about the future. It is something a hamster does. It is a method the species developed in the past to live well now. During life, death exists primarily in the future and is not something an animal worries about. It is busy living, until its life stops. Consequently, in the old rules of man, death was not prominently present. Language created the future as an entity and with it death as the moment the future ends. Without the old rules, death also became much more present because it was used as a means of imposing the new rules, as will be argued below. Being conscious of a future death did not fit into a religion based on the old rules and was therefore given a separate place situated after life: the afterlife [49–51], with heaven and later hell [52]. Hell was an attempt to force the religious rules on people by fear. Heaven and hell are constructs that did not originate in the old rules, but from the new world that emerged when language began to shape life.

## 7. Punishment

### 7.1. Punishment and the law

The concept of punishment is specifically human. Animals do not punish. They hit back or defend themselves or they correct their young with a tap or a peck (see e.g. [53]). But this is always a restricted measure and happens instantaneously so that the relationship with the event is completely clear. Punishment and revenge are mainly human peculiarities brought about by language and the chaos that arose after the old rules disappeared as a binding element, as an all-determining factor.

The old rules were obeyed by all humans, like all animals obey the rules of their species. When spoken language destroyed the human rules, new ones were devised through language. However, as these rules lacked the natural power of the old rules, a way to force them onto the members of the group they applied to had to be devised. It was found in institutional punishment. Those who violated the new rules were punished. A variety of punishments developed, ranging from dispelling the offender from the tribe to death and later incarceration, and the most gruesome methods of torture were invented. All of these methods are still employed, which in itself is peculiar as the new rules themselves are largely arbitrary, changeable and different across societies. It is remarkable that, even today, among societies with different cultures, these arbitrary rules are considered natural by most of their members [54–60].

Punishment became an important part of the new life. As the new rules had no natural authority, they were systematically violated. The civil order in new language-based forms of society that then developed had to be enforced by coercion: violation of the new rules was punished. Coercion and punishment are the most horrible outcomes of the emergence of language, not only in that they came into being, but also in that they seemed necessary. And coercion was necessary because the new rules did not reflect people’s feelings, which at first undoubtedly remained very similar to the feelings they had when the old rules were still functioning. The new rules did not have the naturalness of the old rules and were not easily accepted and often defied. And if people could or would not comply with the new rules, punishments followed that were often horrific. And in fact, this is the situation we still find ourselves in today.

### 7.2. Punishment and religion

Coercion and punishment has also been an important part of religion, perhaps because religion was strongly influenced by the new rules or because coercion and punishment were the only way to impose religion [61–63]. In the latter case, the result was the opposite of what would be expected from a system that had to integrate the old rules into the new way of living. The old rules were protective and therefore caring. Everybody knew what to expect from others and the general state was one of safety and security .^8^ The religious rules that emerged after the old rules had fallen away tried to be so too, but were, and still are, extremely coercive and often threatening (see e.g. [60]). The cruelty of the gods then mirrored that of the violent verbal way of living. The behavior of the gods toward each other and towards humans could not deviate too far from normal human life to remain acceptable. And so in religion, the old rules mixed with the violent life which emerged with language.

Apparently, religion translated the various facets of what was the ‘old culture’ in an inappropriate way: the caring was there to a certain extent, but the certainty that others would not deviate from the rules, which was an inherent aspect of the old rules and which made them protective, was substituted by coercion and punishment. And perhaps there was no other way to make people follow the religious rules. As the natural force of the old rules was gone, religion could only be imposed through language, which is never totally convincing. The use of force - threats and punishment - was then probably the only alternative to make religion functional.

## 8. Good and evil

Good and evil are generally assumed to be fundamental and original human qualities. They are ‘the nature of the beast’. However, animals do not know evil and consequently pre-language man did not. The examples usually given of animal cruelty are projections of human behavior. When a cat plays with a mouse, there is no cruelty in it. For a cat, a mouse is something to catch and then to play with. To be aware of the suffering the animal causes, it must be able to project the feelings of the victim onto itself, which it does not, as this is not in its rules. The same holds for e.g. infanticide, which for us seems cruel. But for there to be cruelty, animals necessarily need to realize that their behavior causes the victim to suffer and they must also be able to project the victim’s suffering onto suffering they have experienced themselves.

Evil behavior in humans is unnatural behavior, behavior due to the use of language. Good behavior, on the other hand, must be behavior which has its roots in original human behavior, the old rules. Good is empathy, caring for other human beings. Good is the bond felt with others because they are humans. Good is all our admirable acts associated with being one of the human species, our basic behavior originating millions of years ago. Defined in this way, good as we feel it is not an objective quality applicable to all creatures. It only evokes such feelings in humans because it relates to old human rules. For another species, good might be something completely different.

Evil came into existence when the old rules disappeared and constitutes the acts deviating from or opposing those rules. Evil is not a quality comparable in its nature to good. It is anomalous behavior, unnatural and alarming, and it signifies what a bizarre, aberrant creature man has become. The new rules man devised when he developed spoken language do not have the quality of the old rules, which developed over millions of years during the evolution of man. They are artificial and arbitrary and often evil in themselves. The rules determining human societies are attempts to create a way of living which allows spoken language to be used and at the same time satisfy human nature as represented by the old human rules. Owing to the many problems resulting from the use of spoken language, that endeavor has failed, as it was bound to.

## 9. Vengeance and cruelty

Vengeance developed after the old rules ceased to exist. It is a phenomenon unknown to animals. An animal may hit back at another animal, either from its own species or a predator, but this is a direct reaction to the situation. Afterwards, there are usually no serious feelings of hatred toward the opponent. Vengeance belongs to the feelings humans developed after they destroyed their natural way of living and it belongs to the unnatural aspects of human life, like hatred, deceit and cruelty. They all developed as a result of the way the mostly arbitrary new rules had to be imposed and those accompanying new ways of behavior subsequently became commonly accepted.

These new ways of behavior - generally assumed to be the natural, original way humans behave - are manifestations of the deforming effects of language. Language has created a virtual world which is very different from the world as experienced by animals. This language world - our ‘civilization’ - creates its own behavior, which can be anything at all, as it is not, or need not be, related to the real world or to fundamental human nature.

## 10. The law and free will

The law assumes that people have free will in choosing to follow the rules. The problem is, however, that free will is difficult to define [64–67] and the question is whether free will is universally present, applicable to all creatures, or whether it is a human conception, developed with language use. If free will for a rabbit means that it can choose one leaf or another, then it has free will. If it means that it can decide to kill another rabbit, then it does not have free will. A rabbit does not kill other rabbits; this is not in its rules.

For humans, free will with regard to the law is based on the concepts of truth and facts. Truth in this context means that there are circumstances whose properties or qualities can be known without doubt. They cannot be refuted. However, language does not allow for such a situation. As pointed out before, spoken language as a method of communication is fundamentally inaccurate and ambiguous and it is not possible to express an exact proposition accurately in an ambiguous medium. Consequently, whether or not truth or fact or even objectivity exist [68–71], they cannot be communicated verbally in any exact way and are therefore concepts that should not be used in the context of the law and in society in general. Another, but related, problem occurs when a choice has to be made between an emotion and the law. This choice can only be made when the emotion is expressed accurately in language, which is not possible, as was discussed in parts I and II of this research [1,2].

Free will is the choice between what we want – largely based on our feelings – and what the law allows us, which is based on reason and logic. The latter is language and in practice results in hopeless confusion and arbitrariness. Free will is then used to blame the individual as he fails to function under the existing rules and make the ‘right’ choices.

Animals also make choices – one leaf or another – but their choices fall within their rules. With humans, the choices they can make are no longer related to the old rules because these hardly exist as rules anymore. There exist mainly invented rules and, as a result, humans constantly encounter impossible choices. Free will is the inescapable pressure humans face to choose between the mostly arbitrary rules of the law and feelings about often accidental situations caused by inconsistent language constructions.

## 11. Discussion

That verbal communication can cause problems is generally accepted. That these problems are intrinsic and severe is usually not realized. Nonetheless, with all its flaws, verbal communication is a useful tool and when its limitations are acknowledged, it can be an effective method of transferring information. If language use had been restricted to information transfer from the outset, human society could have been very different. As it is, our ‘civilization’ is built on an indiscriminate use of language and an unfounded belief in its usefulness in all situations in life. Our world has thus become a rough and violent place with often cruel punishment of those who violate the arbitrary regulations and laws. In fact, the most salient effect of the introduction of language on human life has been that it has changed the relative easy and peaceful way of living under the old rules into violent and aggressive forms of living under punitive control.

It is peculiar that in today’s society punishment is accepted as perfectly normal. It is used in children as an essential part of their upbringing and education and is seen as natural and self-evident. It is a tool, but it seems to be more than that; it suggests a logic, but it is also emotional. Retaliation and the concept of retribution emerged after the old rules were lost. Animals may retaliate when they feel attacked, but this is directed at the situation rather than the individual. With humans, retaliation and revenge are primarily aimed at the person and are experienced as natural.

I have not found a rationale for punishing a violation of the rules other than a practical one: to enforce them. The question is whether the rules could have functioned without punishment through a more natural coercion, more in line with the natural system of the old rules. Today’s forms of society are permeated with violence, and a choice in favor of a non-violent solution back then could have created a more humane society.

But peaceful cohabitation without the old, natural rules is probably not possible anyway. That our coercive society is based on threat and punishment, is in all probability an inescapable consequence of the use of language.

A major cause of the problems discussed above is the way humans make their thoughts conscious to themselves through language. Using language in thinking serves no purpose. It is a useless and distorting intermediate step in the cognitive process. The resulting verbal message may deviate significantly from the neural cognitive message, a problem not present in visual thinking. This consequence of verbal thinking must surely have been a factor in the way society developed. As proposed in part II of this research [2], a solution to this problem might be to find ways to prevent children from making the switch to verbal thinking when they are in the process of learning language, or to develop educational approaches that do not repress visual thinking. This would preserve the children’s inborn visual thinking capability while they still acquire the normal language skills. If people could then become better thinkers, society might develop into a more humane place to live.

## Data Availability

There are no additional data and materials associated to this study.

## Notes

1. When I use the word ‘language’, I will mean human language. If it is used as the way animals communicate, I will emphasize the distinction.

2. For a very simplified elucidation of this basic mechanism, imagine an ant who finds an obstacle on its path, say a twig. To continue its journey, it has to find a solution to this problem. Its thought process finds three possibilities: it can walk around the twig, it can climb over it and it can crawl under it. The solution to its problem provided by the neural thought process is then expressed to the ant in conscious images: the direct visual image of the twig on the path and, probably largely muscular, images of walking, climbing or crawling. The first image, the twig, is of a real, physically present situation, while the last images are representations of imaginary cognitive activity. They are situations projected in the future but based on previous experiences.

3. There are two ways spoken language might be derived from the neural cognitive process: it might be a direct translation of the process output, or it might originate from the natural, sensory output. The first option is not likely. Children learn to talk by translating their still conscious sensory representation of their thoughts into language and that probably will remain the way the conversion process into language keeps functioning. During their transition into verbal thinker, their original sensory images will slowly become less and less conscious, making the vocal form appear fundamental.

4. The applicability of verbal communication is generally explained and defended by simple examples. However, explicating a complex phenomenon by extrapolating it into a simple form is generally a flawed approach in science and especially in linguistics. In any discussion or reasoning, the verbal message becomes very complex with sentences having an almost unlimited range of possibly deviating meanings. Such a complex process cannot be modeled on the basis of simple examples. This way of trying to explain complex phenomena in science is something I discussed previously in the context of the widespread use of *homeostasis*, an extremely simple and generally used model of drug tolerance, which cannot describe the essential features of the process in any acceptable way ([72–74]).

5. A theoretical nonambiguous language cannot exist. See note F in the previous paper [2].

6. The term ‘visual thinking’ is generally used to indicate sensory thinking, which uses images from all the senses. However, the visual component in sensory thinking is most prominent and also experienced as such, reason to adopt the nomenclature.

7. Empathic feelings exist in many species (see e.g. [75]. or other work by de Waal for empathy in chimpanzees). It necessarily means that empathy in humans is an old rule; existing before language evolved.

8. Prelanguage man was probably monogamous [76–80]. Consequently, the attitude in a human group must generally have been one of peaceful cohabitation. This is also reflected in the fact that most behavior we generally consider as laudable, such as empathy, has its origin in the old rules (see footnote 7). Other species show different forms of cohabitation, while some practice infanticide. But the behavior of animals living in a group is always known and predictable, which must generally contribute to a sense of safety and comfort.
